# From ileum to endocardium: a case report of Yersinia pseudotuberculosis prosthetic valve infective endocarditis

**DOI:** 10.1093/ehjcr/ytag321

**Published:** 2026-05-08

**Authors:** Grace O’Dea, Carlos Sebastian Gracias, Nasser Monzer, Brendan McAdam

**Affiliations:** Department of Cardiology, Beaumont Hospital, Beaumont Road, Dublin D09V2N0, Ireland; Department of Cardiology, Beaumont Hospital, Beaumont Road, Dublin D09V2N0, Ireland; Department of Medicine, RCSI University of Medicine and Health Sciences, Mercer Street Lower, Dublin D027N77, Ireland; Department of Cardiology, Beaumont Hospital, Beaumont Road, Dublin D09V2N0, Ireland

**Keywords:** Case report, Infective endocarditis, Yersinia species, Prosthetic valve

## Abstract

**Background:**

We report a case of *Yersinia pseudotuberculosis* prosthetic valve infective endocarditis (IE) in a 70-year-old woman. To date, *Y. pseudotuberculosis* IE has not been described in the literature.

**Case summary:**

The patient presented with sepsis and a history of acute gastroenteritis, on a background of a long-standing mechanical mitral valve replacement (MVR) for rheumatic valve disease. Initial blood cultures grew *Y. pseudotuberculosis,* and a *trans*-oesophageal echocardiogram (TOE) demonstrated a mobile lesion suggestive of vegetations on the atrial surface of the MVR. Appropriate intravenous antibiotics were commenced with an improvement in both her serum markers and clinical status. The patient made a full clinical recovery, and follow-up imaging showed complete resolution of valvular lesions.

**Discussion:**

In the setting of acute gastroenteritis among patients with prosthetic valves, clinicians must be vigilant for unusual gastro-intestinal organisms as a cause for infective endocarditis.

Learning points

- This is the first reported case of *Yersinia pseudotuberculosis* causing infective endocarditis. Clinicians should consider non-HACEK gram-negative bacteria in IE with gastrointestinal symptoms
- With targeted antibiotics and close follow-up, patients who are deemed at high risk for surgical intervention for prosthetic valve endocarditis can be treated effectively
- Early recognition of infective endocarditis and a multidisciplinary team approach to management is critical, particularly in more complex cases

## Introduction

Over 100 years after William Osler remarked that ‘Few diseases present greater difficulties in the way of diagnosis than malignant endocarditis,’^1^ infectious endocarditis remains one of modern medicine’s great challenges. Infectious endocarditis, defined by infection of a native or prosthetic valve, the endocardium, or implantable cardiac device,^2^ has evolved over the last number of decades to reflect the ongoing advances in healthcare; we are now observing advanced age, immunosuppression, and nosocomial infection as the predominant factors in disease acquisition and severity.^3,4^  *Yersinia* species are a group of gram-negative bacilli, only three of which are found in human hosts.^5^ The most well-known of these is *Yersinia pestis*, causing the plague; however, *Yersinia enterocolitica* and *Yersinia pseudotuberculosis* are common causes of self-limiting gastroenteritis.^6^ Here, we describe the first reported case of infective endocarditis caused by *Y. pseudotuberculosis* in a patient with multiple comorbidities.

## Summary figure

**Figure:**
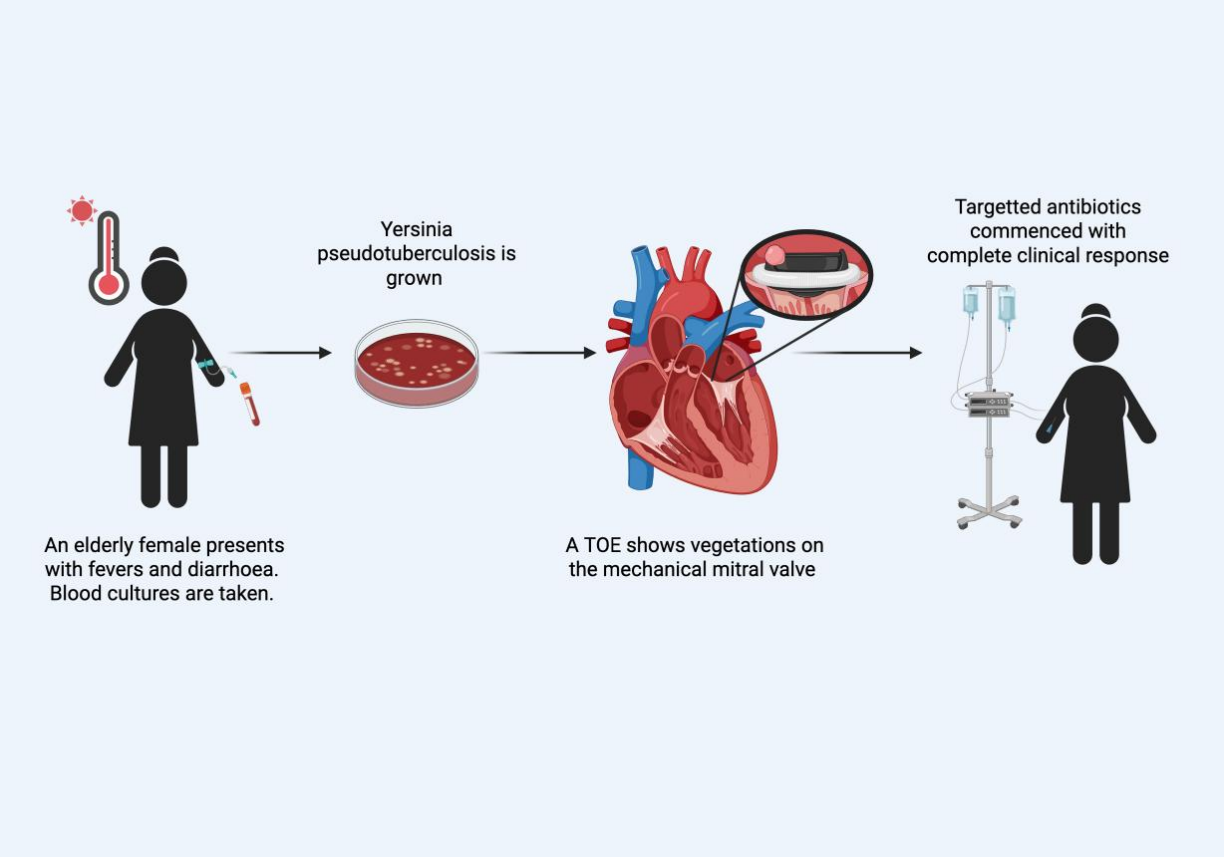
Created in BioRender. O'Dea, G. (2026) https://BioRender.com/xgtt0hc.

## Patient presentation

A 70-year-old woman presented to the emergency department (ED) with a 3-day history of vomiting, diarrhoea, and poor oral intake. She had a background history of a mechanical St. Jude mitral valve replacement (MVR) 7 years prior for rheumatic valve disease with atrial fibrillation, and heart failure with reduced ejection fraction (HFrEF). She also had a history of recurrent falls with a previous subdural haemorrhage while on warfarin and epilepsy. Of note, she had no significant travel history, exposure to fresh water/hikes, and was not an intravenous drug user. She was anticoagulated in the community with therapeutic dose low-molecular weight heparin (enoxaparin 1.5 mg/kg once daily), as she was unable to tolerate warfarin. She was not on any immunosuppressive agents. A *trans*-thoracic echocardiogram (TTE) completed 4 months before her presentation showed a well seated MVR and a left ventricular ejection fraction of 45%.

On presentation to the ED, she was hypotensive (84/50 mmHg), tachycardic (126bpm), and had a temperature of 38.9°C. She was notably drowsy, but physical examination did not yield any positive findings. Initial management in the ED was as per the local sepsis guidelines, with IV piperacillin-tazobactam and IV vancomycin commenced after peripheral blood and stool cultures were sent. She required inotropic support with noradrenaline to maintain an adequate blood pressure, and for this reason, she was admitted to the intensive care unit (ICU).

## Initial investigations

**Figure 1:**
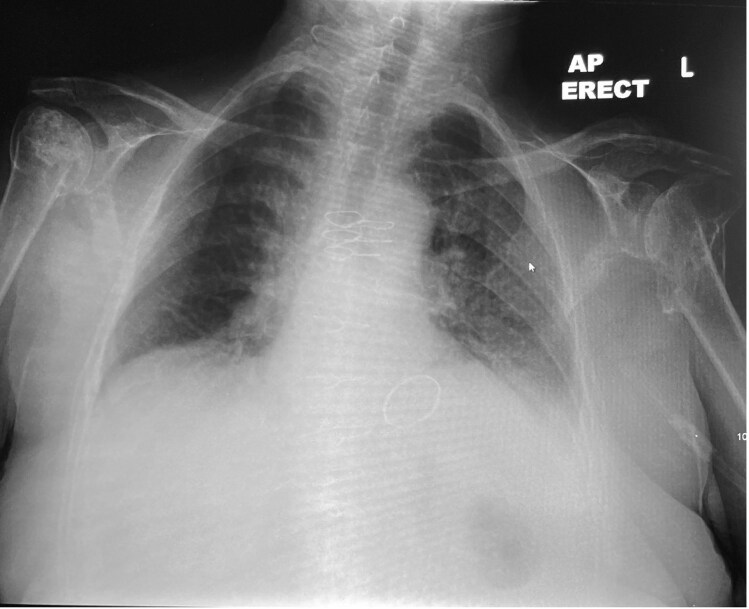
AP erect CXR showing low lung volumes, midline sternotomy wires and a mechanical mitral valve, and old bilateral humerus fractures with extensive degenerative changes.

Initial serological investigations showed raised inflammatory markers with a C-reactive protein (CRP) of 197 mg/L and a white-cell count (WCC) of 16 × 10^9^/L. A chest radiograph on admission showed low lung volumes with no focal consolidation and bilateral old traumatic humerus fractures (*Figure 1*). Subsequent chest radiographs were also clear. Most notably, initial blood cultures cultured gram-negative bacilli after 21 h in the anaerobic bottle—later confirmed to be *Y. pseudotuberculosis.* Stool samples did not isolate any bacteria, including the *Yersinia* species. Mid-stream urine samples were negative. An electrocardiogram (ECG) on admission showed atrial fibrillation with a rapid ventricular response of 126 bpm.

## Management

**Figure 2:**
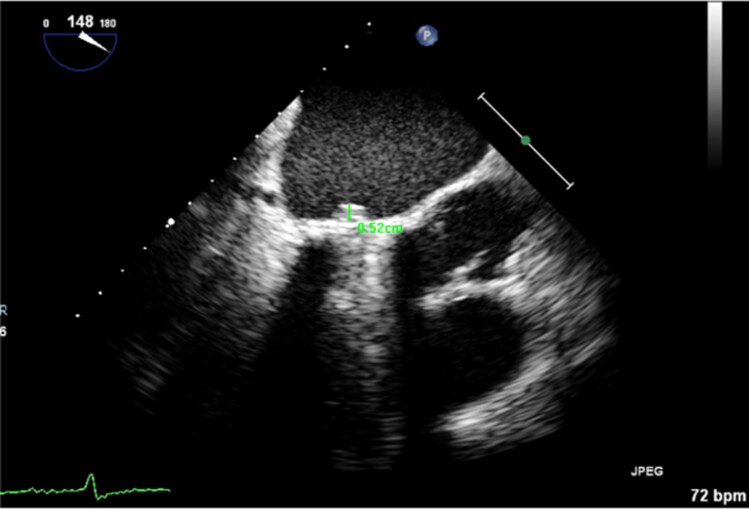
TOE long-axis view with vegetation on the atrial surface of MVR.

**Figure 3:**
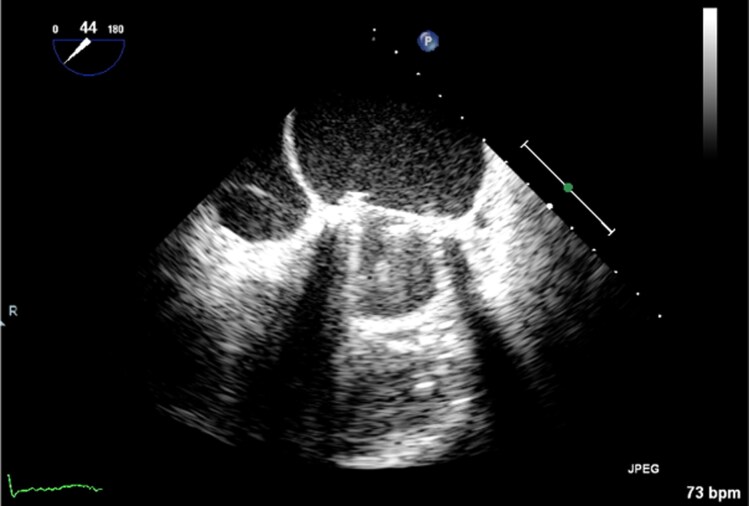
TOE 2 chamber view with vegetation on the atrial surface of MVR.

Following the results of blood cultures, antibiotic therapy was changed to IV cefuroxime and IV gentamicin as per Microbiology recommendations. At this point, she began to have melaena, but haemoglobin remained stable at 11.3 g/dL. An urgent computed tomography (CT) of the abdomen and pelvis showed a small volume of intraperitoneal fluid but no evidence of colitis. On day 3, the pathogen was identified as a *Y. pseudotuberculosis,* sensitive to amoxicillin [minimum inhibitory concentration (MIC) </= 2], and antibiotics were changed accordingly. She was successfully weaned off noradrenaline. A TOE was performed on day 6 when she had stabilized; this showed mobile echo-densities on the atrial surface of the leaflets and mitral valve annulus without valve dysfunction or abscess formation (*Figures 2* and *3*). Given these findings, antibiotics were escalated to IV ceftriaxone (MIC </=1) and IV gentamicin (MIC </=1) in accordance with Microbiology recommendations. The case was discussed at a Heart Team multidisciplinary meeting, and a decision was made for medical management due to high surgical risk due to multiple comorbidities and general frailty. Remarkably, the patient demonstrated significant clinical improvement. She remained afebrile, and her inflammatory markers were normalized. Despite these improvements, on day 8 of admission, she was noted to be confused. A CT brain was completed to assess for septic emboli, and this showed only old bilateral frontal and temporal contusions from previous traumatic injuries. Her confusion improved and was deemed secondary to an infectious delirium. On day 17, gentamicin was discontinued due to labile serum levels. A repeat TOE on day 20 showed interval improvement in the echo-densities adherent to the MVR. Her admission was prolonged due to the need for inpatient rehabilitation with the multidisciplinary team until day 32, at which point, she was discharged home on outpatient intravenous antibiotic therapy (OPAT) to complete 6 weeks of ceftriaxone.

## Follow-up

The patient subsequently presented 3 days post-discharge with two distinct episodes of stool toxin-positive *C. difficile* infection, which was attributed to her prolonged antibiotic course. As a consequence of this, the cephalosporin antibiotics were stopped, and she was first treated with IV metronidazole, tigecycline, and oral vancomycin, whereas on the second occasion, she required oral fidaxomicin. During this period, she underwent a CT abdomen and pelvis, which showed pancolitis. In total, she received 5 weeks of IV ceftriaxone and a follow-up TOE was carried out 8 weeks post-initial presentation, with IE showing further improvement with a reduction in size of the mobile echo-densities attached to the atrial surface of the MVR. The patient made a full recovery and attends regular Cardiology outpatient follow-up. The most recent TTE showed an LVEF of 35%–40% with satisfactory mechanical MVR function and no residual echo-densities attached to the MVR.

## Discussion

To our knowledge, this is the first reported case of *Y. pseudotuberculosis* infective endocarditis involving a mechanical prosthesis and treated successfully with antibiotics without the need for surgery. Within *Yersinia* spp., only three are found in human hosts—*Y. enterocolitica, Y. pestis,* and *Y. pseudotuberculosis.* A recent review of IE caused by *Yersinia* spp. found that of the 12 cases described in the literature, all were *Y. enterocolitica*.^5^  *Y. pseudotuberculosis* is most commonly isolated in cases of gastroenteritis, mesenteric adenitis, or terminal ileitis.^7^ Our suspicion was that the patient had *Y. pseudotuberculosis* gastroenteritis, which progressed to a bacteraemia and then endocarditis through compromise of the intestinal mucosa. Our first learning point in this case is to be vigilant of unusual presentations, such as gastroenteritis, even among an immunocompetent cohort. Second, this case highlights how we should maintain a high suspicion of IE in patients presenting with sepsis with prosthetic valves and the essential role of TOE. Despite having several predictors of poor outcome as per the ESC 2023 Guidelines, namely, older age, frailty, prosthetic valve, and non-HACEK gram-negative infection, our patient recovered well from her initial inpatient course. These predictors of poor outcomes also incur a much higher surgical risk; for this reason, our patient was managed medically, which is contrary to the ESC 2023 Guidelines, which suggest surgical intervention in the setting of prosthetic valve IE. Moreover, this case serves as a reminder that although prolonged IV cephalosporin and aminoglycoside therapy is effective in such cases, physicians should be wary of and monitor patients for subsequent development of *C. difficile* colitis.

## Data Availability

No new data were generated or analysed in support of this research.

## Lead author biography

Dr. Grace O’Dea is currently in her third year of Cardiology Specialty training in Ireland. She is interested in advanced heart failure and cardiac imaging.

## Notes

**Consent:** Consent for writing and publication of this case report was obtained from the patient in accordance with COPE guidelines.
